# Otalgia from Reflex Dental Irritation

**Published:** 1882-09-20

**Authors:** A. G. Hobbs

**Affiliations:** Professor of Eye, Ear and Throat Diseases, In Southern Medical College, Atlanta, Ga.


### Southern Medical Record:

EDITORS:

T. S. POWELL, M.D. W. T. GOLDSMITH, M.D. R. C. WORD, M.D.

*R. C. WORD, M.D., Managing Editor.*

☞All Communications and Letters on Business connected with the RECORD must be addressed to the Managing Editor.

VOL. XII. ATLANTA, GA., SEPT. 20, 1882. No. 9.

### ORIGINAL AND SELECTED ARTICLES.

### OTALGIA FROM REFLEX DENTAL IRRITATION.

BY A. G. HOBBS, M.D.,

Prof. of Eye, Ear and Throat Diseases in Southern Medical College, Atlanta, Ga.

Otalgia from reflex irritation is probably of more frequent occurrence than many have supposed.

Not to mention the commonly observed phenomenon of pain in the ear, accompanying acute affections of the tonsils, it is well known, also, as a not unusual and extremely painful complication of tubercular, cancerous and syphilitic disease of the pharyngeal region.

Otalgia due to malignant or syphylitic disease of the pharynx has often been treated locally for a considerable length of time before the pharyngeal complication has been discovered.

The most remarkable feature of these reflex neuralgic symptoms is that they sometimes occur upon the *opposite* side to the point of irritation.

As an illustration of this feature, I will cite the following case described by Dr. D. B. Delavan, of New York:

"The patient was a finely-developed, well-nourished girl of about twenty, with clear complexion, active circulation and every apparent indication of robust health. Her family history was excellent, and there was no suggestion of any heredity. She had always enjoyed perfect health, with the exception of an attack of double *stitis media*, at the age of seven. At this time there was a purulent discharge from both ears. Since then she has always been slightly deaf in the right ear. The present attack began with a pain in the right ear, which was constant and throbbing, with an occasional paroxysm of lancination, and was much more severe at night. This condition grew worse, producing almost complete insomnia.

"She was first seen four days after the beginning of the attack. Examination with the otoscope revealed both tympani in a healthy condition, with no sign whatever of inflammatory action either of the external or of the middle ear. Rhynoscopic examination failed to discover any cause in the pharynx. The upper teeth were perfect. In the lower jaw the right second molar was slightly carious, while the right wisdom tooth had not yet made its appearance. On the left side the wisdom tooth was through, but the second molar was wanting. Patient stated that several years ago this latter tooth had decayed and come out.

"Applications of heat and anodynes effected no relief. The otalgia was apparently reflex, and seemed due, in all probability, to irritation from the developing wisdom tooth of the right side, or from the carious second molar of the same side.

"The patient was seen in consultation by Dr. Samuel Sexton, who agreed with this hypothesis. At his suggestion she was referred to a competent dentist, who discovered that the deficient second molar of the left side had only lost its crown, while the roots were still impacted in the jaw, although completely covered by mucous membrane. With considerable difficulty three large roots were removed and an abscess of large size was found at the extremity of one of the roots. The wound caused by the removal of these fragments soon healed, and the otalgia quickly disappeared. Since the operation—two months—the carious right second molar has not been filled, nor has the right wisdom tooth yet finished its irruption: nevertheless, there has been no return of the otalgia whatever."

Two cases of reflex dental irritation have occurred in my practice during the last three years. One was a young man about twenty years of age, who presented himself to me with what he called an "earache," which attacked him—usually at night—with lancinating pains that lasted several hours. He had been subject to these attacks for several months. An examination of the ear revealed nothing abnormal; the appearance of the pharyngeal end of the eustachian tube was quite natural; so I was at a loss to know what caused his ear-trouble until, after questioning him closely, he told me that, just before his ear attacks, he always felt a dull, aching pain in a back jaw tooth. I took him to a dentist, who found the crown of a wisdom tooth badly decayed: he extracted it, and the young man told me six months afterward that he had never had a return of the earache. The left ear was affected, and yet the extraction of a decayed right upper wisdom tooth cured the ear. This was a typical case of dental reflex irritation. The other case was a married lady about 36 years of age, who applied to me with an acute catarrhal inflammation of the middle ear. I employed the usual remedies, which should have cured her in a week; but her ear got no better, and the paroxysms of pain became even more frequent and more severe. She suffered with a toothache at the same time. A dentist killed the nerve of the 2d molar tooth and plugged it. I continued the same applications to the ear, and in three days the discharge had ceased, the perforation in the *membrana tympani* had healed and her hearing was entirely restored. The aching tooth in this case was on the same side of the head with the aching ear.

Few instances, if any, have been reported where irritation, not only far removed, but actually upon the side opposite to the neuralgic manifestation, has resulted as in the case cited by Dr. D. and in my first case; and yet, from the almost immediate relief which followed the operation in both instances, there can be no reasonable doubt as to the relation of cause and effect between the dental irritation and the neuralgia.

It would appear, therefore, that a disordered condition of the teeth may exert a powerful influence upon parts with which they seem to have but little nervous connection, and the inference is forced upon us that, in many cases of cephalic neuralgia, the true cause lies in dental irritation, so that relief is not likely to be afforded until the irritation has been removed.

